# Preconditioning with Substance P Restores Therapeutic Efficacy of Aged ADSC by Elevating TNFR2 and Paracrine Potential

**DOI:** 10.3390/biology12121458

**Published:** 2023-11-22

**Authors:** Jiyuan Piao, Hyunchan Cho, Jong Hyun Park, Ki Hyun Yoo, Ildoo Jeong, Hyun Sook Hong

**Affiliations:** 1 Department of Genetic Engineering, Graduate School of Biotechnology, Kyung Hee University, Yongin-si 17104, Republic of Korea; piaojiyuan@khu.ac.kr (J.P.);; 2 Department of Dance, College of Performing Arts & Sport, Han Yang University, Seoul 04763, Republic of Korea; 3 SIMPLE Planet Inc., Seoul 04790, Republic of Korea; 4 Department of Biomedical Science and Technology, Graduate School, Kyung Hee University, Seoul 02447, Republic of Korea; 5 East-West Medical Research Institute, Kyung Hee University, Seoul 02447, Republic of Korea; 6 Kyung Hee Institute of Regenerative Medicine (KIRM), Medical Science Research Institute, Kyung Hee University Medical Center, Seoul 02447, Republic of Korea

**Keywords:** adipose-derived stem cell, TNF-a, TNFR2, substance P

## Abstract

**Simple Summary:**

Stem cell therapy plays a therapeutic role in tissue regeneration in vivo, and its use is increasing for the treatment of incurable diseases. However, stem cells from aged individuals exhibit lower activity, characterized by a high population doubling time and deficient secretion of growth factors. Thus, the transplantation of stem cells with poor activity has shown disappointing results, and we need a strategy to improve the cellular activity of stem cells from aged or diseased individuals. The application of endogenous peptide, substance P, could restore the proliferation rate and paracrine potential of aged stem cells under inflammatory conditions. This suggests the potential use of substance P as a preconditioning factor before stem cell transplantation.

**Abstract:**

Aging leads to a decline in stem cell activity by reducing the repopulation rate and paracrine potential, ultimately diminishing efficacy in vivo. TNF-α can exert inflammatory and cell death actions via Erk by binding to TNFR-1, and survival and tissue repair actions via Akt by binding to TNFR-2. Aged cells are reported to have insufficient expression of TNFR-2, indicating that aged adipose-derived stem cells (ADSCs-E) lack the ability for cell survival and immune control compared to young ADSCs (ADSCs-Y). This study aims to assess the preconditioning effect of SP on the response of ADSCs-E to inflammation. ADSCs-E were treated with SP and then exposed to a high dose of TNF-α for 24 h. Consequently, ADSC-E exhibited weaker viability and lower TNFR2 levels compared to ADSC-Y. In response to TNF-α, the difference in TNFR2 expression became more pronounced in ADSC-E and ADSC-Y. Moreover, ADSC-E showed a severe deficiency in proliferation and paracrine activity. However, preconditioning with SP significantly enhanced the viability of ADSCs-E and also restored TNFR2 expression and paracrine potential, similar to ADSC-Y under inflammatory conditions. Our findings support the idea that preconditioning with SP has the potential to restore the cellular function of senescent stem cells before transplantation.

## 1. Introduction

Stem cells residing in our body play a crucial role in tissue repair and homeostasis in response to injury. Mesenchymal stem cells (MSCs) are adult stem cells that can differentiate into various cell types, including bone, cartilage, and fat. They also generate a sufficient amount of paracrine factors, which accelerate tissue regeneration. The primary sources of MSCs include bone marrow (BM), adipose tissue, cord blood, and dental tissue. The clinical use of MSCs in cellular therapy is steadily increasing worldwide, and efforts to enhance their therapeutic efficacy have been well demonstrated in the treatment of serious diseases.

Aging is an inevitable physiological consequence of living organisms, characterized by complex cellular processes, particularly cellular senescence [1]. This process disrupts stem cell function, leading to a decline in regenerative activity and differentiation potential into specific tissues [2]. Senescent stem cells exhibit low proliferation and viability, mitochondrial deterioration, and insufficient protection against external and internal stress. These alterations contribute to the failure of tissue repair in vivo [1]. Importantly, the cellular activity of bone marrow-derived mesenchymal stem cells (BMSCs) is markedly influenced by background conditions such as aging or disease. Under such circumstances, BMSCs often experience cellular senescence during ex vivo culture, posing challenges in obtaining a substantial quantity of BMSCs for transplantation. Additionally, these cells tend to lose the ability to produce the cytokines and growth factors necessary for tissue repair. Consequently, the transplantation of BMSCs from aged or diseased donors should be approached with caution due to their low repopulation potential and impaired paracrine action. In contrast, adipose-derived stem cells (ADSCs) have garnered attention as an alternative. They are characterized by their abundant distribution, ease of isolation, and rapid re-population in vitro. Furthermore, ADSCs exhibit cellular characteristics similar to those of BMSCs, including repopulation rate, differentiation potential, and secretion of growth factors. These advantages have elevated the significance of ADSCs in both pharmacological and clinical applications.

Nevertheless, it is essential to note that the activity of ADSCs is also subject to the donor’s background, encompassing factors like underlying diseases or aging. Although not to the same extent as observed in BMSCs, ADSCs derived from aged donors display a prolonged population doubling time and inadequate paracrine activity when compared to their counterparts from younger donors [3]. It has been reported that aged ADSCs significantly compromise their ability to support vascular network formation [4,5]. Schipper highlighted that the production, proliferation rate, and pluripotency of ADSCs in young individuals surpass those in elderly individuals [6]. The expression of senescent genes in ADSCs increases with age [7]. These results should be considered before ADSC transplantation. Furthermore, aged ADSCs with reduced activity may encounter pathological or inflammatory environments in vivo after transplantation, potentially leading to rapid cell death before the stem cells can satisfactorily fulfill their therapeutic roles. This is believed to contribute to the low efficacy of stem cell therapy. Therefore, ensuring the sustained presence of transplanted stem cells in vivo is crucial for the therapeutic effects of stem cell therapy. To endure pathological conditions, enhancing the regenerative potential and survival of stem cells before transplantation is essential. Various strategies have been employed to improve stem cell activity during ex vivo culture, including gene transfection, preconditioning with growth factors, exposure to hypoxic conditions, application of physical stimuli, and cultivation in three-dimensional aggregates [8,9,10,11,12,13]. However, achieving satisfactory therapeutic effects remains a challenge [13,14,15,16]. To enhance stem cell activity, it is crucial to thoroughly investigate the cellular state of ADSCs and to identify potential cellular alterations.

Tumor necrosis factor (TNF) is a representative inflammatory factor that is enriched at the site of injury. TNF exerts its effects through two receptors: TNF receptor 1 and 2 (TNFR1 and TNFR2, respectively). It is now recognized that the expression of TNFR2 is more limited than that of TNFR1, which is expressed throughout the body [17]. TNFR2 is expressed in the MSCs, endothelial, neural, and immune cells [17,18,19,20]. The TNF-mediated signaling pathway via TNFR1 primarily drives a pro-inflammatory program, whereas TNF binding to TNFR2 predominantly initiates immune modulation and tissue regeneration [21,22]. One of the main downstream pathways of TNFR2 is the phosphatidylinositol 3 kinase/protein kinase B (PI3K/AKT) pathway, and its ablation results in the failure to activate Akt signaling [23,24,25,26,27]. Importantly, TNFR2 protein levels decrease with age [23], indicating a loss of repair function in aged individuals. Treatment of TNFR2 knockout (KO) BM-MSCs with TNF-α resulted in increased secretion of pro-inflammatory factors, reduced anti-inflammatory factors, and a lack of angiogenic factors [21]. Blocking TNFR2 decreases the regenerative function of MSCs by reducing their colony-forming efficiency and differentiation potential [21,28]. Signaling through TNFR2 in MSCs supports their regenerative functions and provides therapeutic effects for treating inflammatory and autoimmune diseases [26,27]. These findings emphasize the importance of TNFR2 in stem cell therapeutic activities. TNFR2 signaling has the potential to contribute to tissue regeneration after transplantation if sufficiently maintained in aged stem cells.

Substance P (SP) is an endogenous neuropeptide that binds to the neurokinin 1 receptor and is expressed in various cell types, including neuronal, immune, endothelial, skin, and adult stem cells. Systemic injection of SP induces MSC mobilization, allowing them to repopulate the bone marrow, leading to tissue repair [29]. Therapeutic effects of SP-mediated stem cell mobilization have been demonstrated in various diseases. SP is also involved in the induction of regulatory T cells and M2 macrophages to suppress inflammation [30,31,32]. SP modulates AKT/Glycogen Synthase Kinase-3β (GSK-3β) signaling to protect cells and prevent cellular senescence in response to stress, including inflammation and oxidative stress [33,34,35,36]. Given the previously studied roles of SP, it was expected that SP could potentially restore the impaired cellular activity of ADSCs caused by aging, possibly by controlling TNFR2 signaling.

This study aimed to determine whether SP preconditioning could restore the therapeutic effects of aged ADSC. As the cellular response to inflammatory stimuli is important for cell survival and action, TNFR2, a representative signaling receptor related to survival and immune control, was the focus of this study. Before SP treatment, TNFR2 expression in ADSCs from young individuals (20 s; ADSC-Y) and elderly individuals (70 s; ADSC-E) was compared under normal and inflammatory conditions. Subsequently, the preconditioning effect of SP on the activity of elderly ADSCs under inflammatory stress was explored by assessing TNFR2 levels/signaling, viability, and angiogenic potential.

## 2. Materials and Methods

### 2.1. Materials

SP, antibiotic antimycotic solution (AA), and phenylmethylsulfonyl fluoride were purchased from Sigma-Aldrich (St. Louis, MO, USA). An enzyme-linked immunosorbent assay (ELISA) kit for the hepatocyte growth factor (HGF) and vascular endothelial cell growth factor (VEGF) and recombinant human TNF-α was obtained from R&D Systems (Minneapolis, MN, USA). Alpha modified minimum essential Eagle medium (α-MEM) and fetal bovine serum (FBS) were prepared from Gibco (Grand Island, NY, USA). Welgene (Daegu, Republic of Korea) provided 0.25% trypsin-ethylenediaminetetraacetic acid solution and phosphate-buffered saline (PBS). Anti-GAPDH and proliferating cell nuclear antigen (PCNA) antibody were purchased from Abcam (Cambridge, MA, USA), and WST-1 was provided by Roche (Indianapolis, IN, USA). Cell lysis buffer, anti-TNFR2, Akt, phosphorylated Akt, Erk, and phosphorylated Erk antibodies were purchased from Cell Signaling Technology (Danvers, MA, USA).

### 2.2. Cell Culture

Healthy adipose tissues were provided by the Kyung Hee University Medical Center (IRB# 2016-12-022, 2021-01-011) with the written agreement of the donors. Healthy human-derived subcutaneous adipose tissues (young donors: aged 25–26 years old; elderly donors: aged 76–77 years) were collected and washed in saline to remove debris of clot. In total, 1.5 g of adipose tissue was minced and then treated with collagenase (Sigma Aldrich, St. Louis, MO, USA) for 1 h at 37 °C with gentle shaking. The debris was removed by cell strainers (70 micro, SPL Life, Pocheon, Republic of Korea). Stromal vascular fraction was seeded in α-MEM medium supplemented with 10% FBS and 1% antibiotics at 37 °C with 5% CO_2_. The culture medium was replaced with a fresh medium every other day. Passage 2 ADSC was used.

Human cardiac microvascular endothelial cell (HCME, passage 5, Lonza, Basel, Switzerland) was cultured in EGM-2 (Lonza) for tube formation assay. Media were changed every other day. The THP-1 cell line (passage 10, ATCC) was cultured in RPMI-1640 supplemented with FBS 10% and 1% antibiotics.

### 2.3. Preparation of Cell Extracts and Western Blot Analysis

The cells were lysed with lysis buffer (2 mM Tris-HCl (pH 7.5), 15 mM NaCl, 0.1 mM Na_2_EDTA, 0.1 mM EGTA, 0.1% Triton, 0.25 mM sodium pyrophosphate, 0.1 mM beta-glycerophosphate, 0.1 mM Na_3_VO_4_, 0.1 µg/m leupeptin/2 mM PMSF solution). Lysate protein concentrations were determined using a BCA protein assay kit (Thermo Fisher Scientific, Rockford, IL, USA). The cell lysates were denatured in reducing condition and electrophoresed using sodium dodecyl sulfate-polyacrylamide gel electrophoresis and transferred to a PVDF membrane. Blocking of the membrane was performed with 5% skim milk or bovine serum albumin for 1 h. After blocking, the membranes were incubated with the primary antibodies anti-Akt, phospho-Akt, Erk, phospho-Erk, TNFR2, PCNA, HGF, and GAPDH, followed by anti-immunoglobulin G horseradish peroxidase-conjugated secondary antibody. The blots were visualized using chemiluminescence (GE Healthcare, Buckinghamshire, UK). The expression level was quantified using the Image J program (https://imagej.net/ij/).

### 2.4. SP and TNF-α Treatment

ADSCs were seeded and cultured in α-MEM medium containing 10% fetal bovine serum and 1% antibiotics. At 24 h after the media change, SP was added to the ADSC culture in a final dose of 100 nM, one a day for 2 days. As a control for SP or TNF-α treatment, PBS was used as a vehicle. TNF-α was added at 20, 50, and 100 ng/mL.

### 2.5. WST-1 Assay

WST-1 solution was added to each well at 10% the total volume, and the plate was incubated for 90 min at 37 °C in 5% CO_2_. After incubation, the optical density of each well was measured at a wavelength of 450 nm using an EMax Endpoint ELISA Microplate Reader (Molecular Devices, Sunnyvale, CA, USA). Cellular viability in conditioned medium was expressed as a percentage relative to the activity in the control group.

### 2.6. Cytokine Measurement

The ADSCs were seeded in a 6-well plate in α-MEM medium with or without TNF-α or SP. The concentrations of VEGF and HGF in the conditioned medium were analyzed using ELISA kits according to the manufacturer’s instructions. Absorbance was measured at 450 nm using the EMax Endpoint ELISA Microplate Reader. THP-1 was incubated in the mixture of growth medium and conditioned medium of ADSC-E with TNF and SP (1:1) in the presence of LPS (1 µg/mL). Culture supernatant was collected 24 h later and TNF-α/IL-10 was quantified using ELISA (Abcam). Absorbance was measured at 450 nm using the EMax Endpoint ELISA Microplate Reader.

### 2.7. Tube Formation Assay

Matrigel (BD, San Jose, CA, USA) was coated on μ-Slide Angiogenesis (Ibidi, Munich, Germany) and incubated for 30 min at 37 °C in 5% CO_2_. HCMECs (human cardio microvascular endothelial cells, Lonza) were incubated on the Matrigel with conditioned medium of ADSC-E+ TNF-α or SP-treated ADSC-E+ TNF-α for 6 h at 37 °C in 5% CO_2_. The tube structure was observed under phase-contrast microscopy. The quantification of tube formation was analyzed using Image J.

### 2.8. Statistical Analysis

The data are presented as the mean ± standard deviation (SD) of three independent experiments. Statistical significance was determined using a probability value of less than 0.05. For cell viability assays, a two-way ANOVA with Tukey’s multiple comparisons post hoc test was applied. The analysis of protein expression/secretion involved either one-way ANOVA with Tukey’s multiple comparisons post hoc test or t-tests. Significance levels are indicated as follows: * *p* < 0.05, ** *p* < 0.01, *** *p* < 0.001.

## 3. Results

### 3.1. ADSC-E Is More Susceptible to Inflammatory Stress Than ADSC-Y

**Figure 1:**
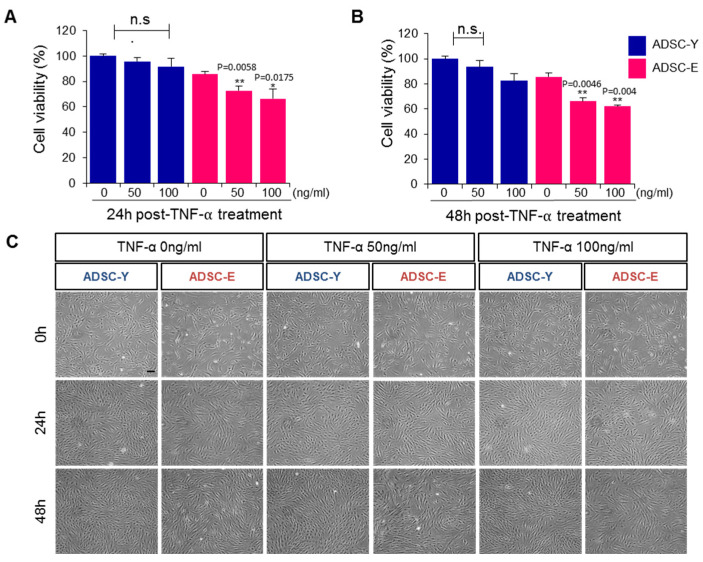
ADSC-E is more susceptible to TNF-α than ADSC-Y. (**A**,**B**) After treating ADSCs with 50 and 100 ng/mL TNF-α for 24 or 48 h, cell viability was evaluated via WST-1 assay (Figure 1A, *p* < 0.0001; Figure 1B, *p* < 0.0001). (**C**) The cellular morphology of ADSCs was observed at 0, 24, and 48 h post-treatment of TNF-α. *p* values of less than 0.05 were considered statistically significant. *: vs. TNF-α 0 ng/mL in ADSC-E. n.s.: non-significant. The data are expressed as the mean ± SD of three independent experiments. The analysis of variation using three-way ANOVA with Tukey multiple comparisons post hoc was performed for statistical analysis. Scale bar: 100 μm. ** *p* < 0.01.

A comparative analysis between ADSC-E and ADSC-Y revealed that ADSC-E exhibited lower viability than ADSC-Y under normal conditions (Figure 1A,C). To assess their response to inflammatory stress, both ADSC-E and ADSC-Y were cultured in the presence of TNF-α. At a concentration of 20 ng/mL, TNF-α did not significantly affect the cell viability of either ADSC-Y or ADSC-E at 24 h (Figure S1). At a concentration of 50 ng/mL TNF-α, ADSC-Y maintained a 90% viability for 48 h, whereas ADSC-E showed a reduction in viability to 72% at 24 h (*p* = 0.0058 vs. TNF-α 0 ng/mL in ADSC-E) and 66% at 48 h (*p* = 0.0046 vs. TNF-α 0 ng/mL in ADSC-E). At a dose of 100 ng/mL TNF-α, ADSC-Y viability decreased to 82% at 48 h, while that of ADSC-E reduced to 66% at 24 h (*p* = 0.0175 vs. TNF-α 0 ng/mL in ADSC-E) and 62% at 48 h (*p* = 0.004 vs. TNF-α 0 ng/mL in ADSC-E) (Figure 1A, *p* < 0.0001 and b, *p* < 0.0001). Observation of cellular morphology showed no distinct alterations in cellular shape between ADSC-Y and ADSC-E cells. However, a comparison of confluency indicated that ADSC-Y proliferated more rapidly than ADSC-E (Figure 1C).

These data suggest that ADSC-E is more susceptible to TNF-α than ADSC-Y, and TNF-α-mediated impairment of ADSC-E occurs within 24 h. Based on this observation, a dose of 50 ng/mL TNF-α was chosen for use in subsequent experiments.

### 3.2. TNF-α Treatment Reduces TNFR2 Expression in ADSC-E, Impairing Repopulation and Paracrine Potentials

**Figure 2:**
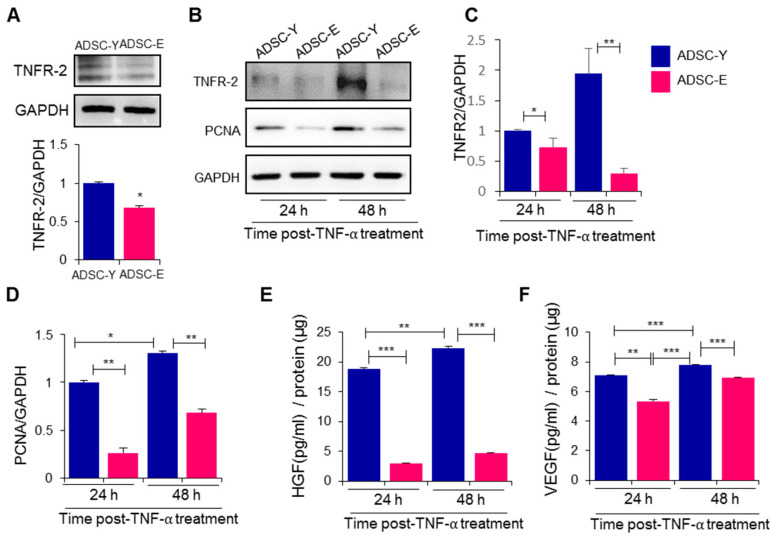
ADSC-E has lower TNFR2 expression than ADSC-Y, leading to impairment of repopulation and angiocrine potential. (**A**) TNFR2 protein level was determined using Western blot, and the expression level was quantified using the Image J program, relative to the GAPDH. T-test was applied for statistical analysis. (**B**–**D**) In the presence of TNF-α, TNFR2 and PCNA protein levels in ADSCs were detected using Western blot analysis, and their expression levels were quantified using the Image J program, relative to the GAPDH. (**E**,**F**) HGF and VEGF concentrations in conditioned media of ADSC were measured using ELISA. *p* values of less than 0.05 were considered statistically significant (* p < 0.05, ** p < 0.01, *** p < 0.001). The data are expressed as the mean ± SD of three independent experiments. Two-way analysis of variation (ANOVA with Tukey multiple comparisons post hoc) was performed for statistical analysis.

The expression of TNFR2 is crucial for stem cell survival under pathological conditions, and its levels have been reported to decrease with aging. To evaluate the impact of aging on TNFR2 expression in ADSCs, we compared TNFR2 levels in ADSC-Y and ADSC-E. TNFR2 expression was found to be lower in ADSC-E than in ADSC-Y cells (Figure 2A, *p* = 0.0174). In response to the potent inflammatory factor TNF-α, ADSC-Y demonstrated an increased TNFR2 expression, while ADSC-E exhibited a decrease (Figure 2A,C, *p* = 0.001). This indicates that ADSC-Y is capable of maintaining cellular viability under inflammatory conditions for tissue repair, whereas ADSC-E struggles to sustain cell survival. PCNA expression increased in ADSC-Y in response to TNF-α, while it remained weak in ADSC-E (Figure 2B,D, *p* = 0.0004). Angiogenesis by ADSCs has been extensively studied, and it constitutes a major aspect of the therapeutic potential of ADSCs. The secretion of HGF and VEGF, representative angiogenic factors, was significantly higher in ADSC-Y compared to ADSC-E in the presence of TNF-α (Figure 2E, *p* < 0.0001; Figure 2F, *p* = 0.001). These results indicate that under inflammatory stress, ADSC-E expresses lower levels of TNFR2, PCNA, and angiogenic factors compared to ADSC-Y. This suggests that ADSC-E may undergo apoptosis and cellular senescence more rapidly than ADSC-Y when exposed to a pathological environment post-transplantation.

### 3.3. Pretreatment with SP Blocks the Loss of Cell Viability in ADSC-E When Exposed to TNF-α, and This Is Accompanied by an Increase in TNFR2 and Angiocrine Factors

**Figure 3:**
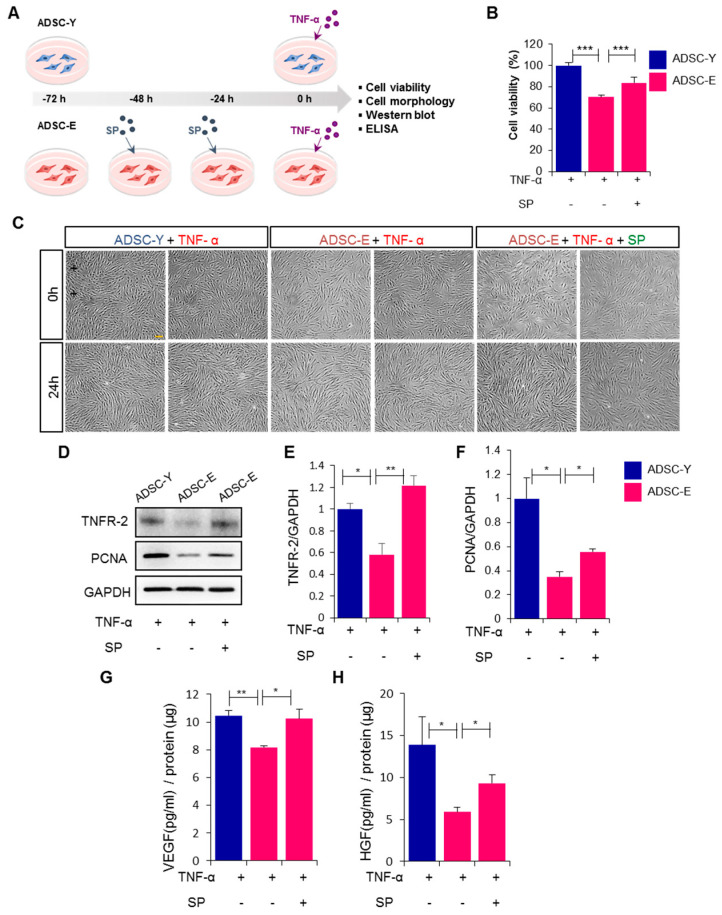
SP preconditioning enhances the viability, repopulation, and angiocrine potential of ADSC-E under inflammatory conditions by elevating TNFR2 expression. (**A**,**B**) After treating ADSC with SP and TNF-α, cell viability was evaluated via WST-1 assay. (**C**) The cellular morphology of ADSC was observed at 0 and 24 h post-treatment of SP and TNF-α. (**D**–**F**) The expression levels of TNFR2 and PCNA on ADSCs were detected using Western blot analysis, and their expression levels were quantified using the Image J program, relative to the GAPDH. (**G**,**H**) VEGF and HGF concentrations in conditioned media of ADSCs were measured using ELISA. *p* values of less than 0.05 were considered statistically significant (* p < 0.05, ** p < 0.01, *** p < 0.001). The data are expressed as the mean ± SD of three independent experiments. Scale bar: 100 μm. One-way analysis of variation (ANOVA) with Tukey multiple comparisons post hoc was performed for statistical analysis.

Under inflammatory stress, ADSC-E exhibited insufficient TNFR2 expression, repopulation capability, and paracrine potential compared to ADSC-Y. This deficiency in ADSC-E may explain its anticipated low efficacy. To restore the cellular function of ADSC-E, we pretreated it with SP and then exposed it to TNF-α to mimic a pathological situation (Figure 3A). While the cell viability of ADSC-E was lower than that of ADSC-Y, SP pretreatment increased the viability of ADSC-E in the presence of TNF-α (Figure 3B).

While the cellular shapes remained similar among the three groups, differences in cellular density over time were observed, possibly due to variations in the repopulation potentials of ADSC-E and ADSC-Y. Notably, SP pretreatment promoted ADSC-E cell proliferation (Figure 3C). Subsequently, we investigated whether the SP-mediated restoration of ADSC-E viability is accompanied by an elevation of TNFR2 and PCNA levels in ADSC-E. When exposed to TNF-α treatment, ADSC-E exhibited lower levels of TNFR2 and PCNA compared to ADSC-Y. Preconditioning ADSCs with SP resulted in the restoration of TNFR2 expression (Figure 3D,E, *p* = 0.0021) and an increase in PCNA expression (Figure 3D,F, *p* = 0.0179). Moreover, SP treatment reversed the deficiency in VEGF and HGF production in ADSC-E (Figure 3G, *p* = 0.0016; Figure 3H, *p* = 0.0091). These findings indicate that preconditioning with SP can effectively restore the cellular function of aging ADSC-E, making them resistant to inflammatory stress. Importantly, the elevation of TNFR2 expression in ADSC-E following SP treatment is likely to contribute to in vivo efficacy by prolonging their survival.

### 3.4. Preconditioning with SP Sustains ADSC-E in a State of Active Akt Signaling, Rather Than Erk Signaling, in Response to TNF-α Treatment

**Figure 4:**
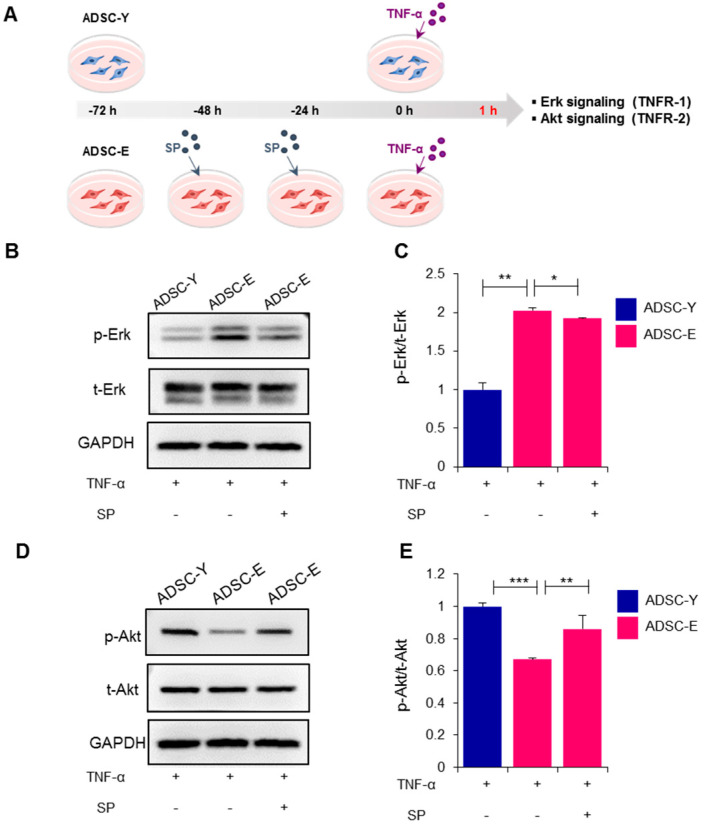
Preconditioning with SP sustains ADSC-E in a state of active Akt signaling, rather than Erk signaling, in response to TNF-α treatment. (**A**) Active levels of Erk and Akt in ADSC-Y, ADSC-E, and SP-treated ADSC-E at 1 h after TNF-α treatment. (**B**,**C**) Phospho-Erk/total-Erk protein level was determined using Western blot, and their expression level was quantified using the Image J program. (**D**,**E**) Phospho-Akt/total-Akt protein level was determined using Western blot, and their expression level was quantified using the Image J program. *p* values of less than 0.05 were considered statistically significant (* p < 0.05, ** p < 0.01, *** p < 0.001). The data are expressed as the mean ± SD of three independent experiments. One-way analysis of variation (ANOVA with Tukey multiple comparisons post hoc) was performed for statistical analysis.

While TNF-α binding to TNFR1 primarily activates Erk, resulting in cell death or inflammatory responses, TNF-α signaling via TNFR2 activates Akt, fostering cell survival, growth, immune regulation, and tissue regeneration. To evaluate the early response to TNF-α, we measured the active levels of Erk and Akt in ADSC-Y, ADSC-E, and SP-treated ADSC-E at 1 h after TNF-α treatment (Figure 4A).

Upon TNF-α treatment, ADSC-E showed higher levels of p-Erk and lower levels of p-Akt compared to ADSC-Y. This discrepancy may be attributed to the inherently lower TNFR2 levels in ADSC-E than in ADSC-Y. However, SP preconditioning restored TNFR2 expression in ADSC-E cells, indicating that SP-treated ADSC-E cells exhibited higher Akt activity than untreated ADSC-E cells. As shown in Figure 4B,C, SP preconditioning induced ADSC-E to exhibit lower levels of phospho-ERK (Figure 4B,C, *p* = 0.0006) and higher levels of phospho-Akt (Figure 4D,E, *p* = 0.0004).

These data suggest that SP preconditioning enhances ADSC-E responsiveness to TNF-α through TNFR2, rather than TNFR1, restoring active Akt signaling for cell survival and growth.

### 3.5. Preconditioning with SP Enhance Paracrine Potential of ADSC-E under Inflammatory Condition

**Figure 5:**
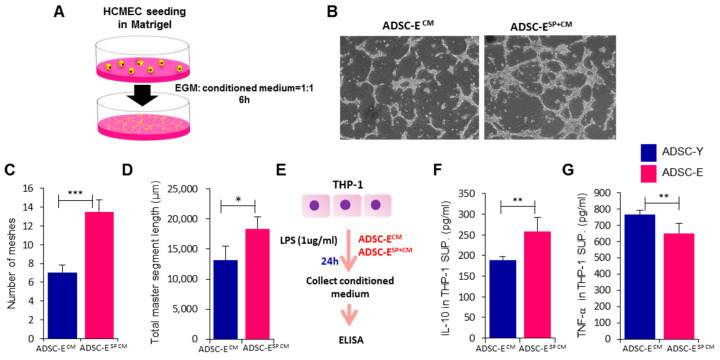
Preconditioning with SP enhances paracrine potential of ADSC-E under inflammatory condition. (**A**) Experimental scheme to evaluate ADSC paracrine potential on in vitro angiogenesis. (**B**) Representative images of HCMECs on Matrigel. (**C**,**D**) Tubular structures formed by HCMECs were quantified by the number of meshes and total master segment length using the Image J program (Scale bar: 200 um). (**E**) Experimental scheme of LPS and conditioned media treatment on THP-1. (**F**,**G**) IL-10 and TNF-α concentrations in conditioned media of THP-1 were measured using ELISA. *p* values of less than 0.05 were considered statistically significant (* *p* < 0.05, ** *p* < 0.01, *** p < 0.001). The data are expressed as the mean ± SD of three independent experiments. SUP., supernatant; ADSC-E^CM^: ADSC-E-conditioned medium; ADSC-E^SP+CM^: SP-pretreated ADSC-E^CM^. Statistical significance was determined using *t*-tests.

The paracrine potential of ADSC was impaired by aging, resulting in a significant decrease in the secretion of crucial angiogenic factors, such as VEGF and HGF, when comparing ADSC-E to ADSC-Y cells. This disparity can impact angiogenesis in injured tissues. To assess the paracrine potential of ADSC, ADSC-E, and SP+ADSC-E, the respective cells were cultured and treated with TNF-α for 24 h. After collecting the conditioned medium (CM), it was added to human cardiac microvascular endothelial cells (HCMEs) on Matrigel. After 6 h, tubular structures were comparatively analyzed (Figure 5A). When comparing the ADSC-E-conditioned medium (ADSC-E^CM^) with the SP-pretreated ADSC-E-conditioned medium (ADSC-E^SP+CM^), the latter induced a more connected structure (Figure 5B). The structure was quantified by examining the number of meshes (Figure 5C, *p* = 0.001) and the total master segment length (Figure 5D, *p* = 0.0152). This restoration is anticipated to contribute to the formation of vascular structures in vitro, as SP treatment elevates VEGF and HGF levels in ADSC-E cells. ADSC secretome has been reported to induce immunosuppressive conditions by promoting the secretion of anti-inflammatory cytokines. In this experiment, the THP-1 human monocyte cell line was stimulated with LPS in the presence of either ADSC-E^CM^ or ADSC-E^SP+CM^ for 24 h, after which the secretion of TNF-α and IL-10 was quantified (Figure 5E). THP-1 cells rarely produced IL-10 in the absence of LPS. As demonstrated in Figure 5F,G, SP-pretreated ADSC-E induced IL-10 production (*p* = 0.0019) and reduced TNF-α secretion (*p* = 0.0047) in THP-1 cells, in comparison to ADSC-E. Therefore, SP treatment may facilitate the generation of immune-modulatory factors from ADSC-E cells.

This result corroborates that SP-mediated restoration of the paracrine potential in ADSC-E cells can contribute to angiogenesis and immune control in an inflammatory environment.

## 4. Discussion

Stem cell transplantation is widely used to treat inflammatory and vascular diseases. Stem cells produce reparative factors and differentiate into injured tissues, thereby facilitating tissue repair in vivo. However, autologous stem cell therapy often requires the use of cellular sources from elderly or diseased individuals, where cellular senescence can occur due to underlying conditions [3,4,5,6]. Senescent stem cells, characterized by weakened cellular activity, are often unable to fulfill their roles post-transplantation, either due to their rapid disappearance or deficient paracrine activity. Studies on ADSCs have increased rapidly, with ADSCs being actively utilized in stem cell therapy owing to their plasticity and ease of collection through minimally invasive surgical techniques. However, the cellular activity of ADSCs is compromised by aging and age-associated pathological conditions. Therefore, the development of novel methods to restore ADSC activity is essential and is expected to significantly enhance the therapeutic efficacy of ADSCs in vivo.

In this study, we investigated differences in ADSC activity between young and elderly individuals. When exposed to the same dose of TNF-α, ADSC-E viability significantly decreased within 24 h, while ADSC-Y maintained viability for 48 h in vitro. This suggests that under inflammatory conditions, ADSC-E may undergo rapid cell death, whereas ADSC-Y may serve as a more resilient cellular therapeutic option. The difference in cell survival rates is likely attributed to survival-controlling factors in ADSCs. TNFR2 signaling, responsible for immune control and cell survival, tends to decrease with aging [23]. Restoration of TNFR2 is closely related to improvements in cellular activity and survival [17,18,19]. In this study, we confirmed the difference in TNFR2 levels between ADSC-Y and ADSC-E. ADSC-E cells, with weak expression of TNFR2, exhibited lower cellular activity compared to ADSC-Y in response to TNF-α. Importantly, ADSC-Y exhibited an increase in TNFR2 levels in response to inflammatory stress, whereas ADSC-E cells failed to maintain TNFR2 levels. This suggests that ADSC-Y enhances survival and regenerative signals during strong inflammation, while ADSC-E loses its survival function, accompanied by a loss of PCNA expression and a decrease in angiocrine factor secretion. ADSCs, with their immune modulatory effects and angiogenic potential, can replace, repair, and regenerate damaged tissues. Consequently, the lack of TNFR2, proliferation activity, and paracrine potential in ADSC-E directly leads to reduced efficacy in stem cell therapy. Therefore, restoring TNFR2 and paracrine potential in ADSCs is a crucial step that must be accomplished prior to clinical application.

To enhance the cell survival and paracrine potential of ADSC-E, we pretreated ADSC-E with SP, followed by TNF-α treatment. This pretreatment with SP significantly restored cellular activity, increased PCNA expression, and boosted VEGF/HGF secretion in ADSC-E under TNF-α stress. Notably, SP-treated ADSC-E exhibited TNFR2 levels higher than those in ADSC-Y. This suggests that preconditioning with SP can alleviate the aging-related loss of TNFR2 in ADSC.

TNF-α can either bind to TNFR1 to activate Erk or to TNFR2 to induce Akt activation. In the early response to TNF-α, SP-treated ADSC-E exhibited higher and lower levels of active Akt and Erk (p-Akt and p-Erk), respectively, compared to non-treated ADSC-E. SP stimulates Akt signaling through PI3K upon binding to NK-1R. This effect can occur independently of TNFR2 signaling. However, since SP was administered to ADSCs before TNF-α in this experimental system, and SP has a very short half-life, higher levels of active Akt (p-Akt) in SP-treated ADSC were inferred to result from the interaction of TNF-α and TNFR2. This indicates that SP preconditioning for 48 h enhances cellular responsiveness via TNFR2, rather than TNFR1, in the presence of TNF-α. The angiogenic potential of SP-treated ADSC-E was further confirmed by the increased secretion of VEGF and HGF, as well as their capacity to form tubular structures in microvascular endothelial cells. The immune modulatory function was assessed by examining IL-10/TNF-α production in THP-1 cells. SP-treated ADSC-E induced an anti-inflammatory response in THP-1 cells, characterized by elevated IL-10 levels and decreased TNF-α levels compared to ADSC-E. These results confirm that SP preconditioning provides ADSC-E with the ability to promote angiogenesis and suppress immune response under conditions of strong inflammation.

In this experiment, SP was administered twice to enhance cellular activity, as a single treatment rarely showed a positive effect on ADSC-E (Figures S2 and S3). Since SP is susceptible to degradation in serum-containing conditions, repeated treatments may be necessary to achieve the desired effects. Importantly, SP is not typically administered with stem cells, and pre-clinical safety studies are not required for its usage in this context. Based on our results, it is presumed that a twice-repeated addition of SP before cell collection for transplantation is appropriate.

This study emphasizes that ADSC impairment can occur due to aging-related factors. However, the loss of stem cell activity is not exclusive to aging and can also be observed in young individuals experiencing acute or chronic inflammation due to factors such as physical activity or obesity. Considering the beneficial role of SP in stem cell activity, these issues can potentially be addressed through SP preconditioning. Therefore, the use of SP is expected to extend to various applications.

## 5. Conclusions

Collectively, preconditioning with endogenous peptide, SP, before transplantation is expected to sufficiently restore the therapeutic activity of ADSC with low TNFR2 and insufficient paracrine potential from diverse donors.

## Data Availability

The datasets used and/or analyzed in the current study are available from the corresponding author upon reasonable request.

## Supplementary Materials

The following supporting information can be downloaded at: https://www.mdpi.com/article/10.3390/biology12121458/s1, Figure S1: The lower-dose of TNF-α does not impair ADSC-E viability; Figure S2: The effects of one-time SP treatment on ADSC-E morphology changes under inflammatory conditions; Figure S3: The effect of one-time SP treatment on ADSC-Y cellular viability and Akt/Erk signaling. Original western blot images of Figure 2, Figure 3 and Figure 4 can be found in the supplementary materials.
