# Supplementary material for: Preconditioning with Substance P Restores Therapeutic Efficacy of Aged ADSC by Elevating TNFR2 and Paracrine Potential
Source: Biology (Basel). 2023 Nov 22;12(12):1458. doi: 10.3390/biology12121458 (PMC10740808; doi:10.3390/biology12121458)
Supplement: Supplementary file 1 [file biology-12-01458-s001.zip › biology-2684610-supplementary.pdf]

Supplementary Figure S1

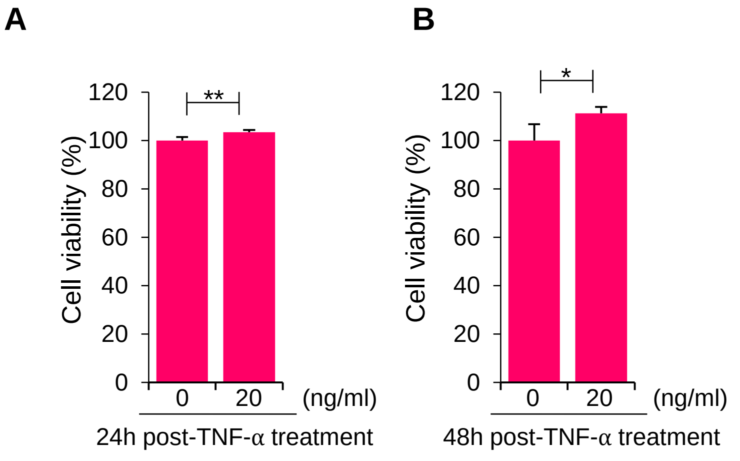

Supplementary Figure S1. The lower-dose of TNF-α does not impair ADSC-E viability

A. After treating ADSC-E with 20 ng/ml TNF-α for 24 hours, cell viability was evaluated by WST-1 assay. B. After treating ADSC-E with 20 ng/ml TNF-α for 48 hours, cell viability was evaluated by WST-1 assay. *p* values of less than 0.05 were considered statistically significant (**p* < 0.05, ***p* < 0.01). The data are expressed as the mean ± standard deviation (SD) of three independent experiments.

Supplementary Figure S2

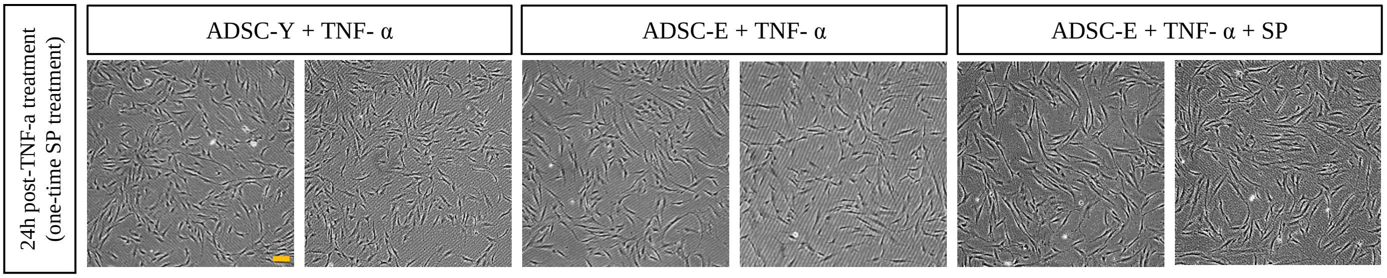

Supplementary Figure S2. The effects of one-time SP treatment on ADSC-E morphology changes under inflammatory conditions.

ADSC-E was treated with SP and 24 later, exposed to TNF-α . The cellular morphology of ADSCs was observed at 24 h post-treatment of TNF-α. Scale bar: 100 μm.

Supplementary Figure S3

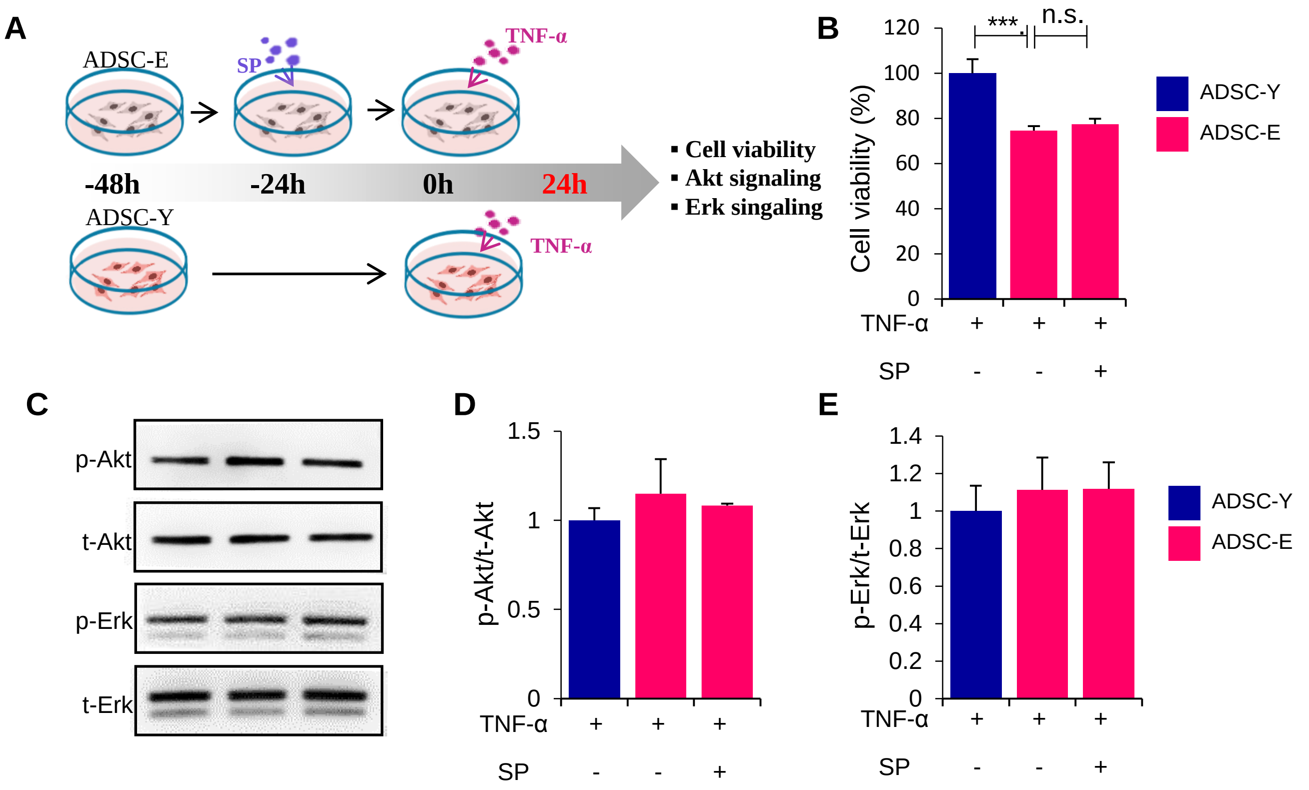

Supplementary Figure S3. The effect of one-time SP treatment on ADSC-Y cellular viability and Akt/ Erk signaling.

(A) Experimental scheme for SP and TNF-α treatment. (B) After treating ADSC with SP and TNF-α, cell viability was evaluated by WST-1 assay. (C-E) p-Akt/t-Akt and p-Erk/t-Erk protein levels were detected by Western blot analysis, and their expression level was quantified using the Image J program. *p* values of less than 0.05 were considered statistically significant (****p*<0.001). The data are expressed as the mean ± standard deviation (SD) of three independent experiments.
