# Supplementary figures and images for: Preconditioning with Substance P Restores Therapeutic Efficacy of Aged ADSC by Elevating TNFR2 and Paracrine Potential
Source: Biology (Basel). 2023 Nov 22;12(12):1458. doi: 10.3390/biology12121458 (PMC10740808; doi:10.3390/biology12121458)

**Fig 2A**

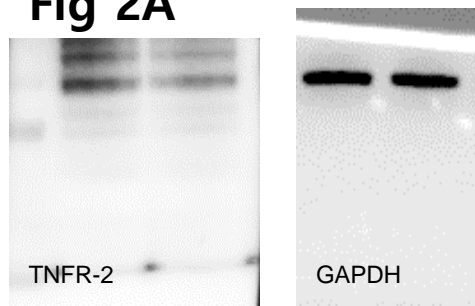

**Fig 3D**

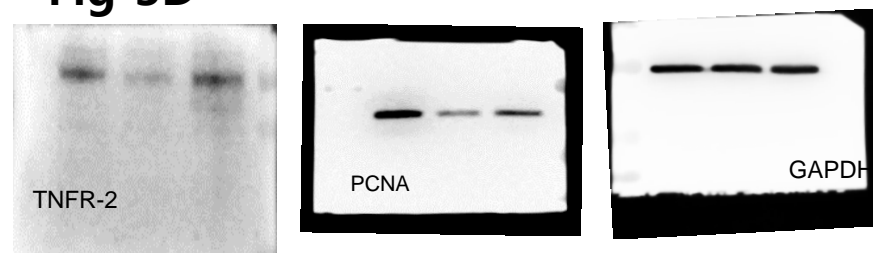

**Fig 2B**

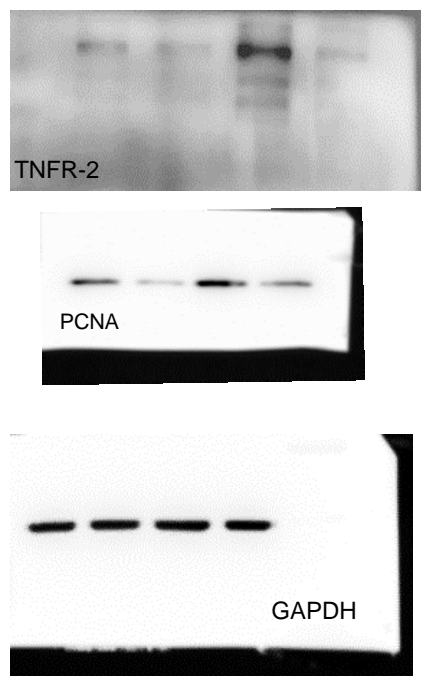

**Fig 4D**

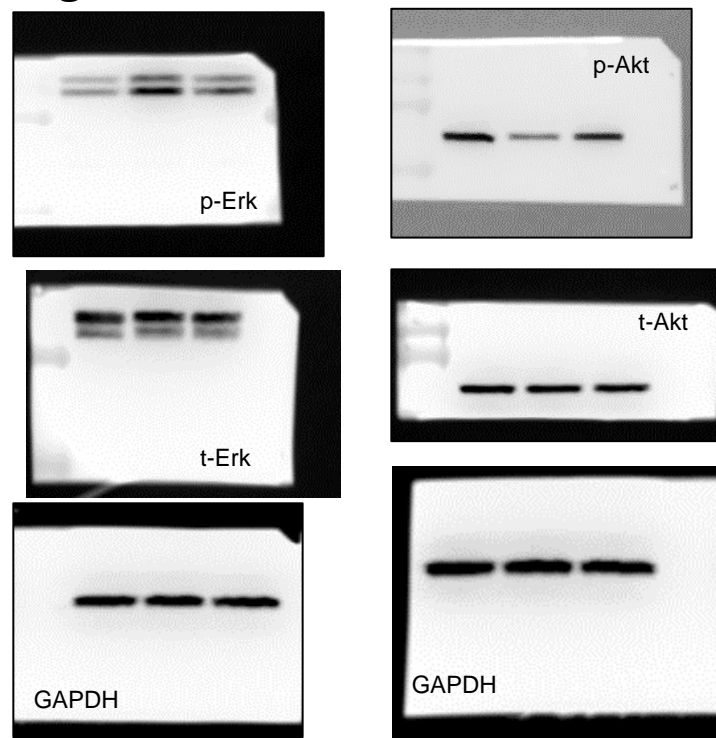

# Supplementary Figure S3

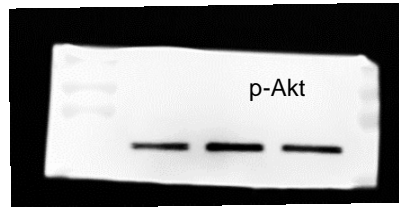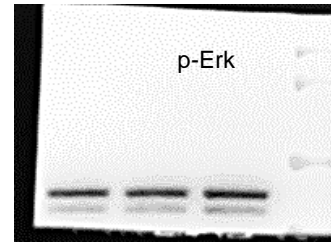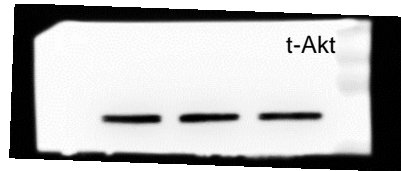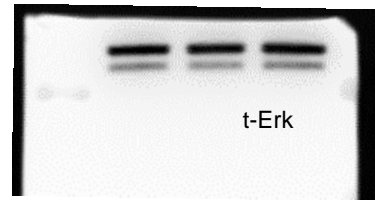

Supplement: Supplementary file 1 [file biology-12-01458-s001.zip › biology-2684610-original wb.pdf]
